# Phospholipid Ether Analogs for the Detection of Colorectal Tumors

**DOI:** 10.1371/journal.pone.0109668

**Published:** 2014-10-06

**Authors:** Dustin A. Deming, Molly E. Maher, Alyssa A. Leystra, Joseph P. Grudzinski, Linda Clipson, Dawn M. Albrecht, Mary Kay Washington, Kristina A. Matkowskyj, Lance T. Hall, Sam J. Lubner, Jamey P. Weichert, Richard B. Halberg

**Affiliations:** 1 Division of Hematology and Oncology, Department of Medicine, University of Wisconsin-Madison, Madison, Wisconsin, United States of America; 2 Department of Oncology, University of Wisconsin-Madison, Madison, Wisconsin, United States of America; 3 Department of Medical Physics, University of Wisconsin-Madison, Madison, Wisconsin, United States of America; 4 Division of Gastroenterology and Hepatology, Department of Medicine, University of Wisconsin-Madison, Madison, Wisconsin, United States of America; 5 Cellectar Biosciences, Madison, Wisconsin, United States of America; 6 Department of Pathology and Vanderbilt-Ingram Cancer Center, Vanderbilt University School of Medicine, Nashville, Tennessee, United States of America; 7 Department of Pathology and Laboratory Medicine, University of Wisconsin-Madison, Madison, Wisconsin, United States of America; 8 Department of Radiology, University of Wisconsin-Madison, Madison, Wisconsin, United States of America; University of North Carolina School of Medicine, United States of America

## Abstract

The treatment of localized colorectal cancer (CRC) depends on resection of the primary tumor with adequate margins and sufficient lymph node sampling. A novel imaging agent that accumulates in CRCs and the associated lymph nodes is needed. Cellectar Biosciences has developed a phospholipid ether analog platform that is both diagnostic and therapeutic. CLR1502 is a near-infrared fluorescent molecule, whereas ^124/131^I-CLR1404 is under clinical investigation as a PET tracer/therapeutic agent imaged by SPECT. We investigated the use of CLR1502 for the detection of intestinal cancers in a murine model and ^131^I-CLR1404 in a patient with metastatic CRC. Mice that develop multiple intestinal tumors ranging from adenomas to locally advanced adenocarcinomas were utilized. After 96 hours post CLR1502 injection, the intestinal tumors were analyzed using a Spectrum IVIS (Perkin Elmer) and a Fluobeam (Fluoptics). The intensity of the fluorescent signal was correlated with the histological characteristics for each tumor. Colon adenocarcinomas demonstrated increased accumulation of CLR1502 compared to non-invasive lesions (total radiant efficiency: 1.76×10^10^ vs 3.27×10^9^ respectively, p = 0.006). Metastatic mesenteric tumors and uninvolved lymph nodes were detected with CLR1502. In addition, SPECT imaging with ^131^I-CLR1404 was performed as part of a clinical trial in patients with advanced solid tumors. ^131^I-CLR1404 was shown to accumulate in metastatic tumors in a patient with colorectal adenocarcinoma. Together, these compounds might enhance our ability to properly resect CRCs through better localization of the primary tumor and improved lymph node identification as well as detect distant disease.

## Introduction

Colon cancer is the second leading cause of cancer-related death in the United States [1]. To effectively treat patients with non-metastatic colorectal cancer, the primary tumor has to be resected with adequate margins and lymph nodes identified for proper staging. Studies have demonstrated that overall survival decreases if fewer than 12 lymph nodes are histologically sectioned [2]–[4]. An important reason for this correlation is that the best indicator for the need for adjuvant chemotherapy for colorectal cancer is the presence of lymph node metastases [5], [6]. An imaging agent that would allow for easier detection of the primary tumor and the regional lymph nodes could have a dramatic impact on the successful treatment of localized colorectal cancers.

(FVB×B6) F1 mice carrying Gt(ROSA)26Sor^tm7(Pik3ca*,EGFP)Rsky^, Tg(Fabp1-Cre)1Jig and *Apc^Min^* provide an informative new model of colorectal cancer with an array of tumors [7]. These mice express a constitutively active form of phosphoinositide 3-kinase (PI3K) in the distal small intestine and colon [8], [9]. We have demonstrated that this dominantly active form of PI3K in the murine intestine causes serrated hyperplasia and advanced neoplasia [10]. These mice also have a germline mutation in the *Adenomatous Polyposis Coli* gene (*Apc^Min/+^*). We also demonstrated that the loss of APC and activation of PI3K synergize causing a significant increase in tumor number, size, and progression in the intestine [7]. In this model, a range of tumors at different stages of development were observed including small adenomas, adenomas with high-grade dysplasia, intramucosal carcinomas and adenocarcinomas penetrating through the serosal layer of the intestine. In addition, metastatic deposits within the regional lymph nodes and mesenteric implants were often observed.

**Figure 1:**
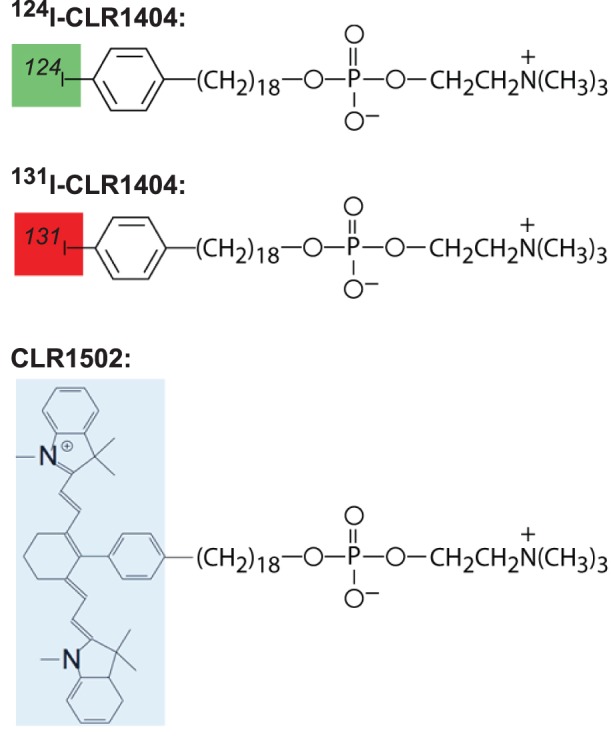
A novel diapeutic platform is based on phospholipid ether analogs. Cellectar Biosciences has developed a phospholipid ether analog-based platform that is currently being investigated for diagnostic and therapeutic oncologic applications. ^124^I-CLR1404 is currently under clinical investigation as a positron emission tomography (PET) tracer. ^131^I-CLR1404 is a therapeutic agent imaged by single photon emission computed tomography (SPECT) and CLR1502 is a near-infrared fluorescent labeled phospholipid ether analog being investigated for regional imaging applications.

Cellectar Biosciences is developing a phospholipid ether analog platform for diagnostics and therapeutics (Fig. 1). The phospholipid ether backbone can be labeled with various imaging markers or therapeutic moieties [11]. CLR1502 is labeled with a near-infrared fluorescent marker. Other members of this platform, including CLR1404, have been shown to accumulate in multiple types of advanced cancers in preclinical models [12], [13]. These agents take advantage of the abundance of cholesterol-rich lipid rafts that are more abundant in cancer cells [14]. These lipid rafts are microdomains within the plasma membrane, which serve to spatially organize signaling pathways important for cellular proliferation and survival [15]. These phospholipid ether analogs take advantage of these lipid rafts as ports of entry into tumors [13]. ^124^I-CLR1404 and ^131^I-CLR1404 are currently being investigated as a positron emission tomography (PET) imaging tracer and as a therapeutic agent imaged by single photon emission computed tomography (SPECT) in clinical trials, respectively. Here, we sought to determine whether CLR1502 accumulates in colorectal cancers, differentiates between invasive and non-invasive intestinal tumors, and detects mesenteric metastatic disease and regional lymph nodes in mouse model of colon cancer. We also tested whether ^131^I-CLR1404 accumulates in metastatic lesions in a patient with colon cancer.

## Methods

### Mouse Husbandry

All animal studies follow the recommendations of the American Association for the Assessment and Accreditation of Laboratory Animal Care (AAALAC). The Institutional Animal Care and Use Committee at the University of Wisconsin-Madison authorized protocols for every animal study accomplished. Female C57BL/6-Gt(ROSA)26Sor^tm7(Pik3ca*,EGFP)Rsky^/J mice (from The Jackson Laboratory, Stock Number 012343), were crossed to male C57BL/6J *Apc^Min^*/J mice (from The Jackson Laboratory, Stock number 002020). Male progeny carrying Gt(ROSA)26Sor^tm7(Pik3ca*,EGFP)Rsky^ and *Apc^Min^* from that first cross were crossed to female FVB/N-Tg(Fabp1-Cre)1Jig mice (from the NCI Mouse Repository, Strain number 01×D8). Progeny of this second cross have a homogenous (FVB×B6)F1 genetic background; those carrying Gt(ROSA)26Sor^tm7(Pik3ca*,EGFP)Rsky^, Tg(Fabp1-Cre)1Jig, and *Apc^Min^* were selected for use in this study. Maintenance and genotyping were performed as previously described [8], [9], [16].

### Administration of CLR1502 and Imaging

Mice between 50 and 60 days of age were selected for study enrollment. CLR1502 was administered at a dose of 50 µg per mouse via injection into the retro-orbital sinus. Mice were sacrificed 96 hours after CLR1502 administration by CO_2_ asphyxiation. Preclinical investigations with CLR1404 demonstrated increased specificity at the 96 hour time-point compared to short intervals. The small bowel and colon were removed, flushed with PBS, split lengthwise, and splayed out. During necropsy, intestinal tumors were visualized with the Fluobeam (Fluoptics; Grenoble, France) near-infrared fluorescent handheld imager. Following removal of the intestines, tumors were then imaged with the IVIS Spectrum imager (Perkin Elmer, Waltham, MA). Excitation and emission wavelengths were 745 nm and 800 nm, respectively. Images were analyzed with Living Image software. Regions of interest (ROIs) were indicated by encircling each area of interest including tumors, Peyer's patches, and normal intestinal epithelium. Radiant efficiency, which is emission light (photons/sec/cm^2^/str) divided by excitation light (µW/cm^2^), was measured. Total (sum of signal intensity across entire ROI), average (average of signal intensity at any one point in the ROI), and maximum (greatest signal intensity at any one point within the ROI) radiant efficiencies were recorded for each region of interest. Measurements were controlled for exposure time, binning, F/stop, subject height, and focus area. Statistical analysis was performed using a two-sided Wilcoxon rank sum test.

### Histology

Following imaging, tissues were rinsed with ethanol and fixed in 10% buffered formalin for 24 hours. Tissues were then stored in 70% ethanol, processed, embedded in paraffin, and sectioned. Every tenth section was stained with hematoxylin and eosin (H&E) for histological review. Two board-certified pathologists reviewed slides independently of each other (M.K.W. and K.A.M.). The imaging results were then correlated with the histology. To make certain that the radiant efficiency of each tumor could be correlated with the histology at the time of necropsy, each tumor identified was assign a number based on the identification of the mouse and location of the tumor. The presence of each tumor was logged and each was photographed prior to imaging. Immediately following necropsy, the intestines were imaged and the ROIs for each tumor were assigned the corresponding number for that tumor. When processing the samples for histology the location of each specific tumor in every block was catalogued so that the identity of each tumor could be maintained.

### Human Subjects and SPECT Imaging Protocol

A patient with treatment-refractory advanced colon cancer was administered ^131^I-CLR1404 as part of a protocol approved by the Health Sciences Institutional Review Board at the University of Wisconsin-Madison (NCT01495663). The patient signed informed consent prior to enrollment, including permission to publish investigational images. ^131^I-CLR1404 was injected intravenously at a dose of 64 mCi. ^131^I emits a principal gamma photon of 364 keV (81% abundance) with a physical half-life of 8.04 days. It also emits beta particles with maximum and mean energies of 0.61 MeV and 0.192 MeV, respectively. First, emission SPECT images were acquired on the GE Infinia SPECT/CT camera using a high energy, parallel hole collimator with counts from the 15% energy window at 364 KeV, with a matrix size of 128×128. A composite image of 120 projections was acquired over 360° with an acquisition time of 30 s/frame and an angular step of 3°. After SPECT acquisition, a CT scan was acquired with a low-dose, helical CT scanner. The CT parameters were 140 KeV and 5 mAs, and no intravenous iodinated contrast was administered. The CT data were used for attenuation correction. The images were reconstructed with a conventional iterative algorithm, ordered subset expectation maximization (OSEM). A workstation providing multiplanar reformatted images was used for image display and analysis.

## Results

### Does CLR1502 accumulate in intestinal tumors?

**Figure 2:**
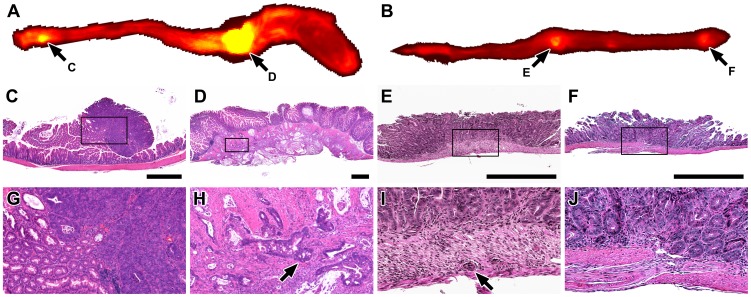
Intestinal tumors demonstrate accumulation of CLR1502. The entire colon (A) and the distal segment of the small intestine (B) were removed at necropsy 96 hours after administration of 50 µg of CLR1502 per mouse. Areas of increased signal intensity were observed using the IVIS Spectrum (excitation 745 nm and emission 800 nm). These areas were found to be non-invasive (colon C and G; distal small intestine F and J) and invasive (colon D and H; distal small intestine E and I) tumors. C, D, E and F are magnified×in G,H, I and J, respectively. Arrows point to malignant glands within the musculature of the intestine. Scale bars C, D, E and F = 1 mm.

To determine whether CLR1502 accumulates in intestinal tumors, mice were injected intravenously with CLR1502. A total of 17 mice (11 male and 6 female) were enrolled in this study. These mice were selected randomly. Based on prior studies, all mice were expected to possess tumors with most mice developing multiple lesions with the distal small intestine and colon (7). The median age was 66 days with a range of 56–139 and the median weight was 24.65 g with a range of 19.62–37.86 g. All mice were injected with 50 µg of CLR1502. Mice tolerated this agent well without acute changes in activity or baseline weight. After 96 hours, mice were sacrificed. The intestines were prepared and imaged using the Fluobeam and IVIS Spectrum systems (Fig. 2; Table S1). All of the mice had intestinal tumors. A total of 38 tumors were evaluated. These tumors ranged from adenomas to intramucosal carcinomas to invasive adenocarcinomas with some of these lesions penetrating through the serosal surface.

**Figure 3:**
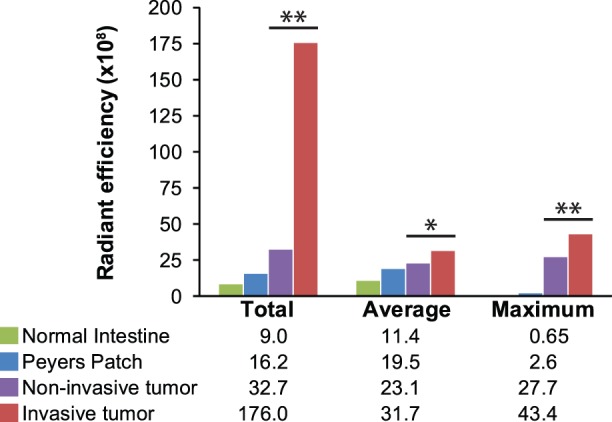
CLR1502 accumulates to a greater extent in invasive compared to non-invasive tumors. Radiant efficiencies were measured of specific regions of interest including normal intestine (n = 16), Peyer's patches (n = 19), non-invasive tumors (n = 7) and invasive cancers (n = 31) using the IVIS Spectrum. A statistically significant increase in total, average and maximum radiant efficiency was seen in the invasive cancers compared to the non-invasive tumors and normal intestinal tissues. * p value <0.05, ** p value <0.01.

CLR1502 is fluorescent in the near-infrared range (excitation 745 nm and emission 800 nm). Accumulation of this fluorophore can be measured by quantifying the radiant efficiency detected. Radiant efficiency, which is emission light divided by excitation light, was used to compensate for non-uniform excitation light patterns. The total, average, and maximum radiant efficiency was measured for tumors, Peyer's patches, and regions of normal mucosa of the small intestine and colon. The measured radiant efficiencies were then correlated with the histology of each region of interest (Fig. 2 and 3). A statistically significant increase in total (invasive 1.76×10^10^ versus mean non-invasive 3.38×10^9^; p = 0.006), average (mean invasive 3.17×10^9^ versus mean non-invasive 2.31×10^9^; p = 0.037), and maximum (mean invasive 4.34×10^9^ versus mean non-invasive 2.77×10^9^ versus; p = 0.009) radiant efficiency was noted in the invasive adenocarcinomas as compared with the non-invasive tumors and normal tissues of the intestine (Fig. 3). All tumors clearly demonstrated accumulation of CLR1502 above that seen in surrounding normal tissue as the total, average and maximum mean radiant efficiency were 9×10^8^, 1.14×10^9^, and 6.5×10^8^, respectively.

### Does tissue thickness account for the increased fluorescence seen in invasive adenocarcinomas?

**Figure 4:**
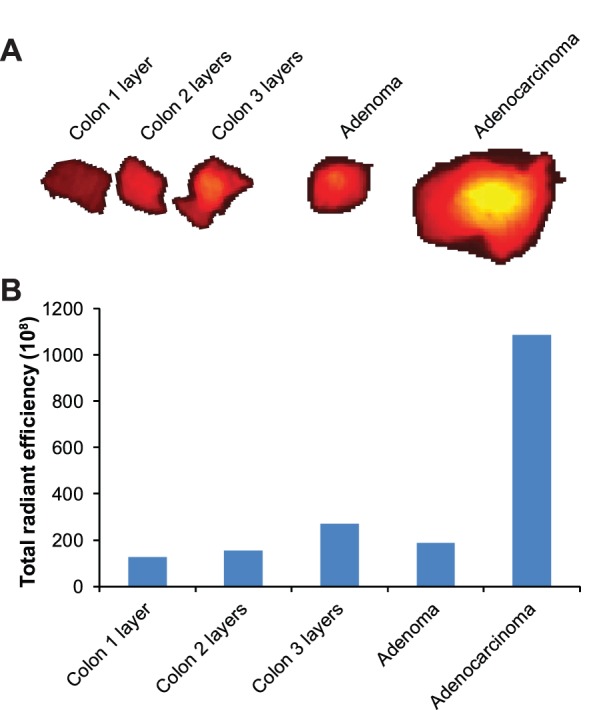
Tumor thickness does not account for the increased signal intensity noted in the intestinal cancers. Necropsy was performed 96 hours post injection of mice with 50 µg of CLR1502 per mouse. To examine the effect of tissue thickness, sections of normal appearing colon were layered upon each other. The radiant efficiency was measured to compare signal intensity between one, two, and three layers of normal colon and intestinal tumors. Note that one layer of normal colon is approximately 1 mm thick. Tissue thickness might account for the increased intensity seen in the adenomas, but does not account for the differences seen with the adenocarcinomas.

Typically, invasive adenocarcinomas were much thicker than the normal tissue or non-invasive tumors. To determine whether the increased signal from the invasive cancers was related to tissue thickness, sections of normal colonic epithelium were layered on top of each other and imaged with the IVIS Spectrum. Radiant efficiency increased with increasing thickness (Fig. 4). The signal intensity of the adenomas and Peyer's patches could be simply explained by an increase in tissue thickness, but signal intensity in the adenocarcinomas cannot be accounted for by tissue thickness alone. Frozen sections of tumors were also analyzed and confirmed an increase in radiant efficiency in the invasive cancers compared to benign tissue (Fig. S1).

### Does CLR1502 accumulate in metastatic disease to the mesentery and in regional lymph nodes?

**Figure 5:**
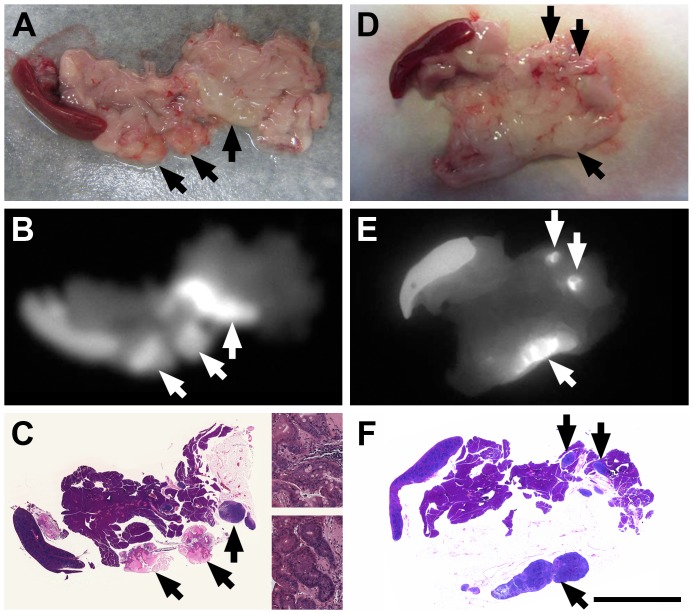
CLR1502 accumulates in metastatic tumor deposits and in regional lymph nodes. Mice underwent necropsy 96 hours after injection with CLR1502. Following removal of the intestine, the mesenteric fat, pancreas, and spleen were isolated as one specimen en bloc. In one of the mice, two metastatic tumor deposits measuring 4 mm in size were noted within the mesentery (A). These lesions were easily visualized with the Fluobeam hand-held near-infrared imager (B). These lesions were confirmed to be metastatic malignant lesions on H&E (C). A higher magnification H&E stained section of each tumor deposit is shown as inserts in panel C. Regional lymphadenopathy (D) was also shown to accumulated CLR1502 using the Fluobeam (E). Lymph nodes as small as 2 mm in size were easily detectable. No malignant cells were observed within these hyperplastic lymph nodes (F). Arrows point to tumor and uninvolved lymph nodes that accumulated CLR1502. Size bar F = 1 cm.

The mice used in this study can develop metastatic disease to the mesenteric adipose tissue. One of the mice treated with CLR1502 was found to have metastatic disease to the mesentery at necropsy. After the intestine was removed, the mesenteric adipose tissue, pancreas, and spleen were removed and splayed out (Fig. 5A). The mesenteric tissue was then imaged with the Fluobeam (Fig. 5B). This tissue was then fixed in formalin and sectioned. Histology confirmed the presence of metastatic cancer in two locations (Fig. 5C). Both of these areas demonstrated accumulation of CLR1502.

CLR1502 was also shown to accumulate in the regional lymph nodes. The mesenteric tissue including lymph nodes was removed and splayed out from six mice. A representative example is displayed in Fig. 5D. These tissues were evaluated with the Fluobeam and increased signal intensity was noted in the mesentery correlating with mesenteric lymphatic tissue including lymph nodes as small as 1–2 mm in diameter (Fig. 5E). Histology revealed that lymphatic tissue was hyperplastic without any evidence of cancer involvement (Fig. 5F).

CLR1502 may be used to interrogate the resection cavity for remaining lymphatic tissue or tumor after resection of the surgical sample. After removal of the intestine and mesenteric tissue from a CLR1502-treated mouse, occult retroperitoneal lymph nodes were visualized with the Fluobeam (Fig. S2A–B). These lymph nodes were only 2 mm in size. Resection of these lymph nodes was confirmed by demonstrating their absence in the surgical cavity and presence within the resected specimens (Fig. S2D–E).

### Do phospholipid ether analogs accumulate in human colorectal cancers?

**Figure 6:**
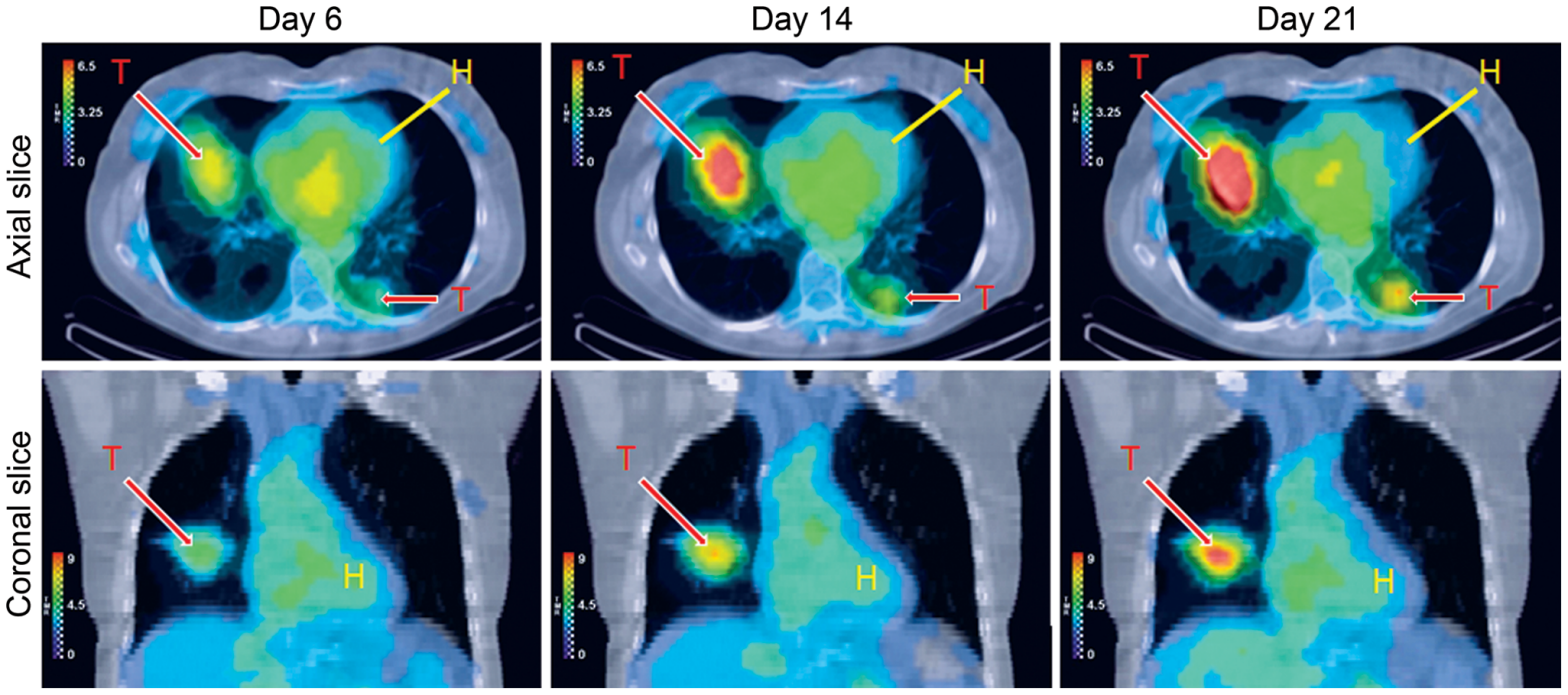
^131^I-CLR1404 accumulates in a patient with metastatic colon cancer. As part of a phase I clinical trial, SPECT images were acquired between 6 and 21 days after administration of ^131^I-CLR1404. Areas of increased signal intensity corresponded to known areas of metastatic colon cancer in both lungs. Prolonged retention of this agent within these lesions was also demonstrated. T, tumor; H. heart.

^131^I-CLR1404 is currently under investigation as a therapeutic agent. Patients with treatment-refractory solid malignancies have been treated with ^131^I-CLR1404. A patient with metastatic colorectal cancer was administered 64 mCi of ^131^I-CLR1404. SPECT imaging demonstrates accumulation and prolonged retention of ^131^I-CLR1404 within metastatic colorectal cancer in the lung (Fig. 6). The primary tumor was no longer present in this patient. This image is representative of the uptake seen in patients with colorectal cancer in this on-going study. No significant toxicities have been described in patients treated with CLR-1404 at doses used for imaging studies.

## Discussion

Despite advances in screening and prevention, colorectal cancer still remains a prevalent and deadly disease. Patients with localized colorectal cancer undergo resection of the primary tumor with the associated mesenteric tissue, which can be a curative procedure. Resection of the primary tumor with adequate surgical margins is necessary to optimize the patient's chance at cure. Many colon tumors are detected prior to the tumor penetrating through the muscularis propria or serosa, making them more difficult to identify at the time of the operation. Tattooing of the colon at the site of the primary tumor is often done to increase the likelihood of removing the tumor with adequate margins.

In addition to resection of the primary tumor with adequate margins, the number of regional lymph nodes evaluated for cancer has been shown to be an important prognostic marker [2]–[4]. Patients in whom less than 12 lymph nodes were evaluated were shown to have a decreased overall survival compared to patients having 12 or more. This is likely related to under-staging of the cancer. The presence of regional lymph node involvement indicates a significantly greater risk of cancer recurrence [17]. The current standard of care is to offer adjuvant chemotherapy for patients with lymph node positive disease [5]–[6]. To identify the lymph nodes for pathological review, the lymph nodes have to be dissected out of the resection specimen. This task can be difficult and onerous with metastases to lymph nodes frequently being less than 5 mm in diameter. An imaging agent that would allow for the more efficient identification and isolation of these lymph nodes would have great potential to increase our ability to adequately stage colon cancers.

Here, we demonstrate that CLR1502 can be used for the detection of colon cancer tissue within the mammalian intestine and also for the improved isolation of regional lymph nodes. This agent holds great potential to aid in the detection of the primary tumor to allow resection with sufficient margins. This surgery could be accomplished laparoscopic or robotically with a near-infrared fluorescent scope. In addition, a hand held imager, such as the Fluobeam, could greatly improve our ability to detect lymphatic tissue or free tumor deposits within the mesentery. We also present here that another compound with the same backbone structure, ^131^I-CLR1404, accumulates in the tumors of a patient with metastatic colorectal cancer. This result is important for multiple reasons including the potential benefit of ^131^I-CLR1404 as a therapeutic, ^124^I-CLR1404 as a PET imaging tracer, and for the translation of the results presented here using CLR1502 as a near-infrared fluorescent imaging agent. Future studies will examine the use of these agents in patients with multiple cancer types and we are excited about the possibility of their use for cancer treatment, whole body imaging, and surgical guidance.

## Supporting Information

Figure S1 **Tumor thickness does not account for the increased signal intensity noted in the intestinal cancers.** Frozen sections of intestinal tissues were obtained from mice without treatment (A) or after 96 hours post treatment with 50 µg of CLR1502 per mouse (B). These sections were imaged with the IVIS Spectrum. Increased signal intensity was observed in the adenocarcinoma (1) compared to other benign intestinal sections (2–5). Increased signal intensity was also observed corresponding to lymphatic tissue in an intestinal segment (LN).
(EPS)

Figure S2 **CLR1502 can be used to identify lymphatic tissue for resection.** A mouse 96 hours post injection with 50 µg of CLR1502 per mouse was examined with the Fluobeam hand-held imager after removal of the intestine and mesenteric tissue (A). Examination with the Fluobeam identified additional remaining retroperitoneal lymph nodes (B). These were excised and no longer visible in the retroperitoneum with the Fluobeam (C). *Ex vivo* the specimens were confirmed to retain the Fluobeam signal and were found to be 2 mm lymph nodes (D).
(TIF)

Table S1 **Radiant efficiencies of CLR1502 treated tumors and benign tissues.** Intestinal tissues from *FC^1^3K^1^Apc^Min/+^* mice 96 hours after administration of 50 µg per mouse of CLR1502 were imaged with the IVIS Spectrum (excitation 745 nm and emission 800 nm). Radiant efficiencies were measured of specific regions of interest including normal intestine, Peyer's patches, non-invasive tumors and invasive cancers.
(DOCX)
