# Supplementary figures and images for: Phospholipid Ether Analogs for the Detection of Colorectal Tumors
Source: PLoS One. 2014 Oct 6;9(10):e109668. doi: 10.1371/journal.pone.0109668 (PMC4186834; doi:10.1371/journal.pone.0109668)

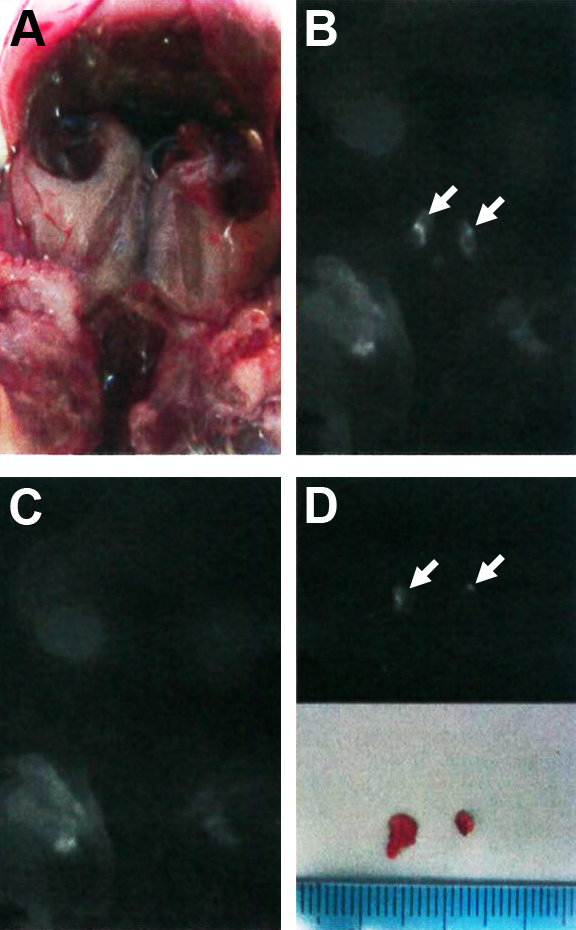

Supplement: Figure S2 — CLR1502 can be used to identify lymphatic tissue for resection. A mouse 96 hours post injection with 50 µg of CLR1502 per mouse was examined with the Fluobeam hand-held imager after removal of the intestine and mesenteric tissue (A). Examination with the Fluobeam identified additional remaining retroperitoneal lymph nodes (B). These were excised and no longer visible in the retroperitoneum with the Fluobeam (C). Ex vivo the specimens were confirmed to retain the Fluobeam signal and were found to be 2 mm lymph nodes (D). (TIF) [file pone.0109668.s002.tif]
