# Supplementary material for: Phospholipid Ether Analogs for the Detection of Colorectal Tumors
Source: PLoS One. 2014 Oct 6;9(10):e109668. doi: 10.1371/journal.pone.0109668 (PMC4186834; doi:10.1371/journal.pone.0109668)
Supplement: Table S1 — Radiant efficiencies of CLR1502 treated tumors and benign tissues. Intestinal tissues from FC13K1ApcMin/+ mice 96 hours after administration of 50 µg per mouse of CLR1502 were imaged with the IVIS Spectrum (excitation 745 nm and emission 800 nm). Radiant efficiencies were measured of specific regions of interest including normal intestine, Peyer's patches, non-invasive tumors and invasive cancers. (DOCX) [file pone.0109668.s003.docx]

**Supplementary Table S1** **Radiant efficiencies of CLR1502 treated tumors and benign tissues.** Intestinal tissues from *FC^1^3K^1^Apc^Min/+^* mice 96 hours after administration of 50µg per mouse of CLR1502 were imaged with the IVIS Spectrum (excitation 745nm and emission 800nm). Radiant efficiencies were measured of specific regions of interest including normal intestine, Peyer's patches, non-invasive tumors and invasive cancers.

| **Sample ID** | **Animal ID** | **Age (days)** | **Gender** | **Histology** | **Total radiant efficiency [p/s] / [µW/cm²]** | **Average radiant efficiency [p/s/cm²/sr] / [µW/cm²]** | **Maximum radiant efficiency** |
| --- | --- | --- | --- | --- | --- | --- | --- |
| 1 | 10196 | 139 | Female | Invasive | 1.24E+10 | 2.03E+09 | 2.99E+09 |
| 2 | 10196 | 139 | Female | Invasive | 5.07E+10 | 2.55E+09 | 3.99E+09 |
| 3 | 10196 | 139 | Female | Invasive | 4.42E+10 | 2.05E+09 | 3.99E+09 |
| 4 | 10196 | 139 | Female | Invasive | 9.33E+09 | 1.86E+09 | 2.57E+09 |
| 5 | 11028 | 66 | Male | Invasive | 5.92E+10 | 4.39E+09 | 7.23E+09 |
| 6 | 11028 | 66 | Male | Invasive | 3.17E+09 | 2.51E+09 | 2.97E+09 |
| 7 | 11028 | 66 | Male | Invasive | 2.81E+09 | 2.70E+09 | 3.12E+09 |
| 8 | 11025 | 66 | Female | Invasive | 2.13E+09 | 2.30E+09 | 2.59E+09 |
| 9 | 11025 | 66 | Female | Invasive | 3.61E+09 | 2.92E+09 | 3.18E+09 |
| 10 | 11025 | 66 | Female | Invasive | 3.62E+09 | 2.93E+09 | 3.27E+09 |
| 11 | 11212 | 60 | Male | Invasive | 1.15E+10 | 4.46E+09 | 6.03E+09 |
| 12 | 11212 | 60 | Male | Invasive | 6.12E+10 | 6.44E+09 | 8.86E+09 |
| 13 | 11217 | 60 | Male | Invasive | 3.93E+10 | 4.15E+09 | 5.58E+09 |
| 14 | 11270 | 56 | Male | Invasive | 2.39E+10 | 4.09E+09 | 5.35E+09 |
| 15 | 11270 | 56 | Male | Invasive | 4.17E+09 | 2.84E+09 | 3.22E+09 |
| 16 | 11205 | 62 | Female | Invasive | 1.70E+09 | 2.76E+09 | 3.39E+09 |
| 17 | 11205 | 62 | Female | Invasive | 1.03E+10 | 3.34E+09 | 4.55E+09 |
| 18 | 11205 | 62 | Female | Invasive | 3.93E+10 | 5.31E+09 | 8.54E+09 |
| 19 | 11210 | 62 | Female | Invasive | 1.51E+10 | 2.81E+09 | 3.62E+09 |
| 20 | 11210 | 62 | Female | Invasive | 1.30E+10 | 2.27E+09 | 3.57E+09 |
| 21 | 11210 | 62 | Female | Invasive | 1.10E+10 | 2.41E+09 | 3.67E+09 |
| 22 | 11210 | 62 | Female | Invasive | 8.92E+09 | 2.29E+09 | 3.65E+09 |
| 23 | 11268 | 58 | Female | Invasive | 7.40E+09 | 2.82E+09 | 3.68E+09 |
| 24 | 11276 | 58 | Female | Invasive | 1.55E+10 | 4.19E+09 | 6.73E+09 |
| 25 | 11678 | 73 | Female | Invasive | 6.10E+09 | 3.59E+09 | 4.07E+09 |
| 26 | 11678 | 73 | Female | Invasive | 4.89E+09 | 1.78E+09 | 2.36E+09 |
| 27 | 11680 | 73 | Female | Invasive | 5.46E+10 | 4.81E+09 | 7.27E+09 |
| 28 | 11790 | 66 | Male | Invasive | 3.93E+09 | 3.00E+09 | 3.74E+09 |
| 29 | 11790 | 66 | Male | Invasive | 1.01E+10 | 3.16E+09 | 3.64E+09 |
| 30 | 11785 | 66 | Female | Invasive | 2.83E+09 | 2.22E+09 | 3.09E+09 |
| 31 | 11785 | 66 | Female | Invasive | 9.89E+09 | 3.37E+09 | 4.04E+09 |
| 32 | 11028 | 66 | Male | Non-invasive | 5.00E+09 | 2.88E+09 | 3.32E+09 |
| 33 | 11028 | 66 | Male | Non-invasive | 4.37E+09 | 1.92E+09 | 2.53E+09 |
| 34 | 11680 | 73 | Female | Non-invasive | 3.54E+09 | 3.52E+09 | 4.19E+09 |
| 35 | 11268 | 58 | Female | Non-invasive | 3.27E+09 | 1.73E+09 | 2.08E+09 |
| 36 | 11678 | 73 | Female | Non-invasive | 3.10E+09 | 2.12E+09 | 2.71E+09 |
| 37 | 11783 | 66 | Female | Non-invasive | 1.99E+09 | 1.56E+09 | 1.89E+09 |
| 38 | 11784 | 66 | Female | Non-invasive | 2.42E+09 | 2.41E+09 | 2.69E+09 |
| 39 | 10196 | 139 | Female | Peyer's patch | 6.10E+08 | 1.06E+09 | 1.38E+09 |
| 40 | 11028 | 66 | Male | Peyer's patch | 1.97E+09 | 1.76E+09 | 2.38E+09 |
| 41 | 11028 | 66 | Male | Peyer's patch | 1.52E+09 | 1.29E+09 | 1.78E+09 |
| 42 | 11028 | 66 | Male | Peyer's patch | 1.10E+09 | 1.46E+09 | 1.84E+09 |
| 43 | 11025 | 66 | Female | Peyer's patch | 2.05E+09 | 2.96E+09 | 3.31E+09 |
| 44 | 11025 | 66 | Female | Peyer's patch | 1.79E+09 | 3.10E+09 | 3.52E+09 |
| 45 | 11025 | 66 | Female | Peyer's patch | 1.18E+09 | 1.71E+09 | 2.10E+09 |
| 46 | 11025 | 66 | Female | Peyer's patch | 1.88E+09 | 1.47E+09 | 2.01E+09 |
| 47 | 11025 | 66 | Female | Peyer's patch | 2.75E+09 | 1.74E+09 | 2.23E+09 |
| 48 | 11212 | 60 | Male | Peyer's patch | 1.23E+09 | 3.12E+09 | 3.69E+09 |
| 49 | 11215 | 60 | Male | Peyer's patch | 1.58E+09 | 3.16E+09 | 3.38E+09 |
| 50 | 11215 | 60 | Male | Peyer's patch | 2.30E+09 | 1.99E+09 | 2.54E+09 |
| 51 | 11205 | 62 | Female | Peyer's patch | 1.10E+09 | 1.64E+09 | 1.95E+09 |
| 52 | 11210 | 62 | Female | Peyer's patch | 1.27E+09 | 1.32E+09 | 1.59E+09 |
| 53 | 11268 | 58 | Female | Peyer's patch | 2.24E+09 | 1.38E+09 | 1.92E+09 |
| 54 | 11276 | 58 | Female | Peyer's patch | 1.41E+09 | 1.30E+09 | 1.76E+09 |
| 55 | 11680 | 73 | Female | Peyer's patch | 8.45E+08 | 1.22E+09 | 1.59E+09 |
| 56 | 11790 | 66 | Male | Peyer's patch | 2.21E+09 | 2.72E+09 | 3.15E+09 |
| 57 | 11785 | 66 | Female | Peyer's patch | 1.76E+09 | 2.68E+09 | 2.91E+09 |
| 58 | 11025 | 66 | Female | Normal | 9.22E+09 | 3.79E+09 | 4.27E+09 |
| 59 | 11025 | 66 | Female | Normal | 1.34E+09 | 1.65E+09 | 1.92E+09 |
| 60 | 11025 | 66 | Female | Normal | 1.40E+09 | 1.34E+09 | 1.77E+09 |
| 61 | 11270 | 56 | Male | Normal | 2.50E+10 | 4.00E+09 | 4.73E+09 |
| 62 | 11205 | 62 | Female | Normal | 6.98E+08 | 1.11E+09 | 1.58E+09 |
| 63 | 11205 | 62 | Female | Normal | 1.60E+09 | 2.23E+09 | 2.68E+09 |
| 64 | 11205 | 62 | Female | Normal | 5.08E+08 | 8.50E+08 | 9.27E+08 |
| 65 | 11205 | 62 | Female | Normal | 8.14E+08 | 1.24E+09 | 1.32E+09 |
| 66 | 11210 | 62 | Female | Normal | 6.97E+08 | 1.39E+09 | 1.58E+09 |
| 67 | 11210 | 62 | Female | Normal | 4.65E+08 | 8.61E+08 | 1.11E+09 |
| 68 | 11268 | 58 | Female | Normal | 6.90E+08 | 1.49E+09 | 1.61E+09 |
| 69 | 11268 | 58 | Female | Normal | 4.65E+08 | 1.10E+09 | 1.12E+09 |
| 70 | 11276 | 58 | Female | Normal | 7.00E+08 | 1.30E+09 | 1.36E+09 |
| 71 | 11276 | 58 | Female | Normal | 5.63E+08 | 1.04E+09 | 1.12E+09 |
| 72 | 11680 | 73 | Female | Normal | 1.36E+09 | 7.31E+08 | 8.13E+08 |
| 73 | 11680 | 73 | Female | Normal | 2.75E+09 | 1.42E+09 | 1.64E+09 |
